# Entomopathogenic fungi, *Metarhizium anisopliae* and *Beauveria bassiana* reduce the survival of *Xenopsylla brasiliensis* larvae (Siphonaptera: Pulicidae)

**DOI:** 10.1186/1756-3305-5-204

**Published:** 2012-09-19

**Authors:** Ladslaus L Mnyone, Kija R Ng’habi, Humphrey D Mazigo, Abdul A Katakweba, Issa N Lyimo

**Affiliations:** 1 Biomedical and Environmental Thematic Group, Ifakara Health Institute, P.O. Box 53, Off Mlabani Passage, Ifakara, Tanzania; 2 Pest Management Centre, Sokoine University of Agriculture, P.O. Box 3110, Morogoro, Tanzania; 3 Department of Medical Parasitology and Entomology, Faculty of Medicine, Weill-Bugando University College of Health Sciences, Mwanza, Tanzania, P.O. Box 1464, Mwanza, Tanzania

## Abstract

**Background:**

Entomopathogenic fungi, particularly those belonging to the genera *Metarhizium* and *Beauveria* have shown great promise as arthropod vector control tools. These agents, however, have not been evaluated against flea vectors of plague.

**Findings:**

A 3-h exposure to the fungi coated paper at a concentration of 2 × 10^8^ conidia m^-2^ infected >90% of flea larvae cadavers in the treatment groups. The infection reduced the survival of larvae that had been exposed to fungus relative to controls. The daily risk of dying was four- and over three-fold greater in larvae exposed to *M. anisopliae* (HR = 4, *p*<0.001) and *B. bassiana* (HR = 3.5, *p*<0.001) respectively. Both fungi can successfully infect and kill larvae of *X. brasiliensis* with a pooled median survival time (MST±SE) of 2±0.31 days post-exposure.

**Conclusion:**

These findings justify further research to investigate the bio-control potential of entomopathogenic fungi against fleas.

## Findings

Plague is a zoonotic disease caused by *Yesinia pestis*, one of the most pathogenic bacteria [1]. Plague may occur in three forms: bubonic, septicaemic and pneumonic plague. Without prompt and appropriate treatment, plague, especially the two latter forms, is virtually always fatal. This disease remains an extremely important public health concern in many parts of the world. Strikingly, however, over 90% of all cases and deaths reported to date occur in Africa [2]. The most seriously affected countries are Mozambique, Malawi, Uganda, Madagascar, Democratic Republic of Congo and Tanzania. For example, Lushoto, one of the most active plague foci, plague outbreaks that have occurred from 1980–2003 involved about 7000 reported cases/suspects with over 630 (9%) deaths [3].

Plague can be transmitted from small mammals particularly rodents to humans through flea bites. Among others, common flea vectors include *Xenopsylla cheopis*, *X. brasiliensis, Dinopsyllus lypusus* and *Pulex irritans*[1]. Control of flea vectors remain the mainstay of plague control in many countries. Vector control has greatly contributed in reducing severity of plague transmission and/or outbreaks. However, several problems may be associated with recurring plague. Firstly, drastic climate change and less global commitment to the disease might escalate the risk of plague outbreaks. Secondly, high operational costs as a result insecticides are applied only during and soon after disease outbreaks and yet are not always done promptly and sufficiently. Lastly, the development of insecticide resistance in fleas that in turn threatens sustainability of control strategies. Insecticide resistance in fleas has been reported in Tanzania and globally [4,5]. Insecticide resistance has greatly affected the global efforts to control and possibly eliminate mosquito borne diseases such as malaria [6,7]. The extent of the problem of insecticide resistance in fleas is currently low, however, it would be quite advantageous to learn from the situation in malaria and prevent it from happening in plague. Clearly, immediate efforts to develop effective and cheap complementary flea control strategies are necessary. The aim should be to develop control strategies, which can synergize with the existing ones; and are less likely to suffer from the problem of resistance.

Entomopathogenic fungi, particularly those belonging to the genera *Metarhizium* and *Beauveria* have shown great promise as arthropod vector control tools [8-10]. These fungi are soil-borne and predominantly infect soil dwelling insects [11]. These agents, however, have not been evaluated against flea vectors despite the fact that the latter (especially pre-mature stages) prefer soil microhabitats. Therefore, preliminary study was conducted to demonstrate if *M. anisopliae* and *B. bassiana* can infect and kill larval stages of fleas, *X. brasiliensis*.

Two fungal isolates were used: *Metarhizium anisopliae* ICIPE-30 and *Beauveria bassiana* IMI 391510. Dry conidia of the fungi were suspended in highly refined enerpar M002 oil (BP Southern Africa LTD) to obtain a test solution. A total of 1200 μl of the test solution was applied evenly to a 15 × 25 cm piece of painting paper using a metal bar (0.31 mm diameter; paper and applicator bar from RK Print Coat Instruments, London) [9] giving a uniform concentration 2 × 10^8^ conidia m^-2^. Untreated control replicates used paper with enerpar only. After drying (24 h at room temperature), paper was cut circumferentially to fit inside Petri dishes without scraping the conidia off.

Flea larvae reared at Sokoine University of Agriculture Pest Management Centre insectary (SPMC) were exposed to treated and untreated paper inside a Petri dish in three replicates; 20 larvae per replicate. The larvae were held in the Petri dish for 3 h, after which they were transferred to transparent holding containers containing a mixture of sterile sand and ground dry cow blood. The survival and fungus infection status of larvae were monitored daily for up to 15 d following procedures described elsewhere [8]. They were maintained at 27.5 ± 2.5°C and ≥82% RH. The survival of flea larvae was analyzed by Cox regression analysis, using SPSS version 16. Cox regression generated hazard ratios (HR) indicating the daily risk of dying for larvae in each treatment and control. Kaplan–Meier pairwise method was used to obtain median survival times (MST) for treated and untreated flea larvae.

**Figure 1:**
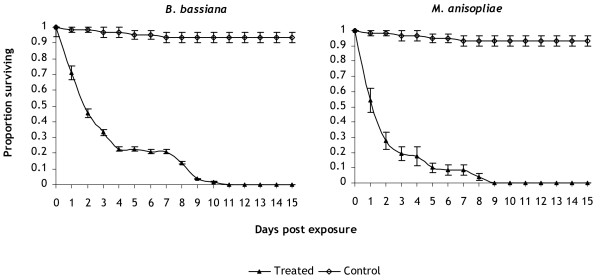
**Survival of *Xenopsylla brasiliensis *larvae after 3 h exposure to entomopathogenic fungi, *Metarhizium anisopliae *ICIPE-30 and *Beauveria bassiana *I93-825.**

*M. anisopliae* and *B. bassiana* were capable of infecting > 90% of *X. brasiliensis* larvae (fungal growth on cadavers observed after incubation for 4–6 d). Both fungal isolates significantly reduced the survival of all exposed larvae compared to controls (Figure 1); 100% mortality in exposed larvae was achieved in 9 d for *M. anisopliae* and 11 d for *B. bassiana*. In the control, >94% of larvae were still alive by 15 d and most had transformed to pupae; and none of the cadavers showed fungal sporulation. For *M. anisopliae*, the daily risk of dying was four-fold greater in exposed larvae relative to their controls (HR = 4, *p* < 0.001). For *B. bassiana*, the daily risk of dying was over three-fold greater in exposed larvae relative to their controls (HR = 3.5, *p* < 0.001). However, the survival of flea larvae exposed to either fungus was equally reduced (*χ*^2^ = 3.45, *p* > 0.05); median survival time (MST ± SE) of larvae exposed to *M. anisopliae* and *B. bassiana* was 2 ± 0.33 d and *B. bassiana* 2 ± 0.31 d respectively. The MST for controls could not be estimated because mortalities did not exceed 50%.

Biological control agents that can effectively be delivered to target flea larvae could be a potential complementary approach to existing control strategies. Here we have shown for the first time that dry conidia of *M. anisopliae* and *B. bassiana* formulated in enerpar oil can infect and kill larvae of *X. brasiliensis*; 100% mortality in exposed larvae was achieved in 9 d for *M. anisopliae* and 11 d for *B. bassiana*. Similar rates of mortality have been reported when fungi were tested against other arthropods [12,13]. Perhaps, most importantly, fungi have multifaceted mode of action and cause delayed mortality. As such, these agents would pose less selection pressure for development of resistance [14] against fleas; and thus offer an alternative to chemical insecticides. Although insecticide resistance in fleas is currently not that alarming, it is important to invest in developing resistance management strategies before the situation worsens. Integrating entomopathogenic fungi with chemicals, could delay resistance development and thus extend the lifetime of insecticidal control strategies [15]. Interestingly, these fungi have been found to equally infect insecticide resistant anopheline mosquitoes [16,17]. This could equally be a possibility in flea vectors. Other advantages of fungi include cost effectiveness [15] and minimum risk to the environment and living organisms [16].

Over 90% of treatment larvae showed fungal growth after death. In view of the nature of fleas’ soil microhabitats, similar infection rates could occur in nature and thus allow dissemination of conidia to uninfected individuals. Uninfected eggs, larvae, pupae and adult fleas may get infected from auto-disseminated conidia from the sporulating cadavers and contaminated microhabitats. Interestingly, all the immature and mature stages of fleas share similar microhabitats at some point, which maximizes the chance of infecting all stages. Flea microhabitats are often cooler, humid and normally protected from direct sunlight (e.g. rodent burrows). Arguably, such environments will allow fungal conidia to remain infective for relatively longer periods. Equally important, self propagation of fungal propagules might be a possibility. The effect of environmental factors on fungal potency is probably the most fundamental challenge that may interfere with the field use of fungi.

We envisage that, the revealed ability of *M. anisopliae* and *B. bassiana* to reduce survival of flea larvae will stimulate research to further investigate the flea control potential of these biological control agents. Studies to fully understand both lethal and sub-lethal effects of fungi on not only flea larvae, but also eggs and adults, are justifiable.

## Competing interests

All authors have no competing interest to declare and all have actively contributed to this study and review.

## Authors' contributions

Conceived and designed the experiments: LLM BSK INL HDM. Performed the experiments: LLM AAK. Analyzed the data: LLM INL HDM. Wrote the paper: LLM INL KRN AAK. Reviewed the paper: BSK CL. All authors read the final version of the manuscript.
